# Supplementary material for: NeoPrecis: enhancing immunotherapy response prediction through integration of qualified immunogenicity and clonality-aware neoantigen landscapes
Source: Nat Commun. 2026 Jan 23;17:1966. doi: 10.1038/s41467-026-68651-6 (PMC12932759; doi:10.1038/s41467-026-68651-6)
Supplement: Supplementary file 1 — Supplementary Information [file 41467_2026_68651_MOESM1_ESM.pdf]

# Supplementary Information for
# NeoPrecis: Enhancing Immunotherapy Response Prediction through Integration of Qualified Immunogenicity and Clonality-Aware Neoantigen Landscapes

Ko-Han Lee[1], Timothy J. Sears[1], Maurizio Zanetti[2,3], Hannah Carter[1,3,4*]

**Affiliations**

[1] Bioinformatics and Systems Biology Program, University of California San Diego, La Jolla, CA, USA

[2] The Laboratory of Immunology, Department of Medicine, University of California San Diego, La Jolla, CA, USA

[3] Moores Cancer Center, University of California San Diego, La Jolla, CA, USA

[4] Department of Medicine, Division of Genomics and Precision Medicine, University of California San Diego, La Jolla, CA, USA

*Correspondence to hkcarter@health.ucsd.edu

This document contains Supplementary Figures 1-14 and Supplementary Tables 1-2.

# Supplementary Figures

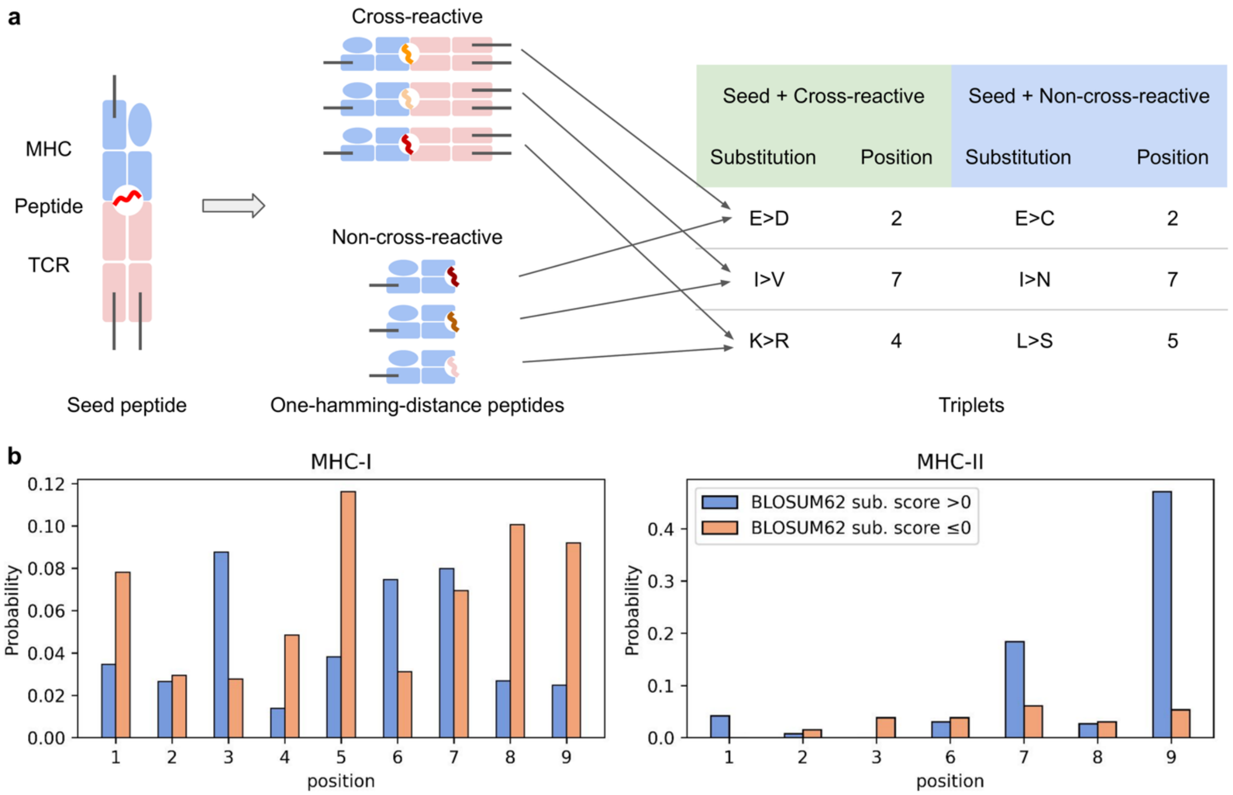


**Supplementary Fig. 1. Preparation and characteristics of the cross-reactive peptide triplet dataset. a** A schematic diagram of the triplet generation. For each TCR-peptide complex serving as the seed, cross-reactive samples were identified by selecting remaining peptides that share the same MHC allele and CDR3 sequence but differ by one Hamming distance in the peptide sequence. For each seed-cross-reactive pair, a non-cross-reactive sample was randomly selected as a peptide with a one-Hamming-distance difference from the seed that was not included in the cross-reactive peptide set. Each triplet consists of a seed, a cross-reactive, and a non-cross-reactive sample. **b** Frequency of amino acid substitutions grouped by BLOSUM62 substitution scores and positions in seed-cross-reactive pairs (mimicking non-immunogenic substitutions) for MHC-I (n = 1,153 pairs) and MHC-II (n = 261 pairs).

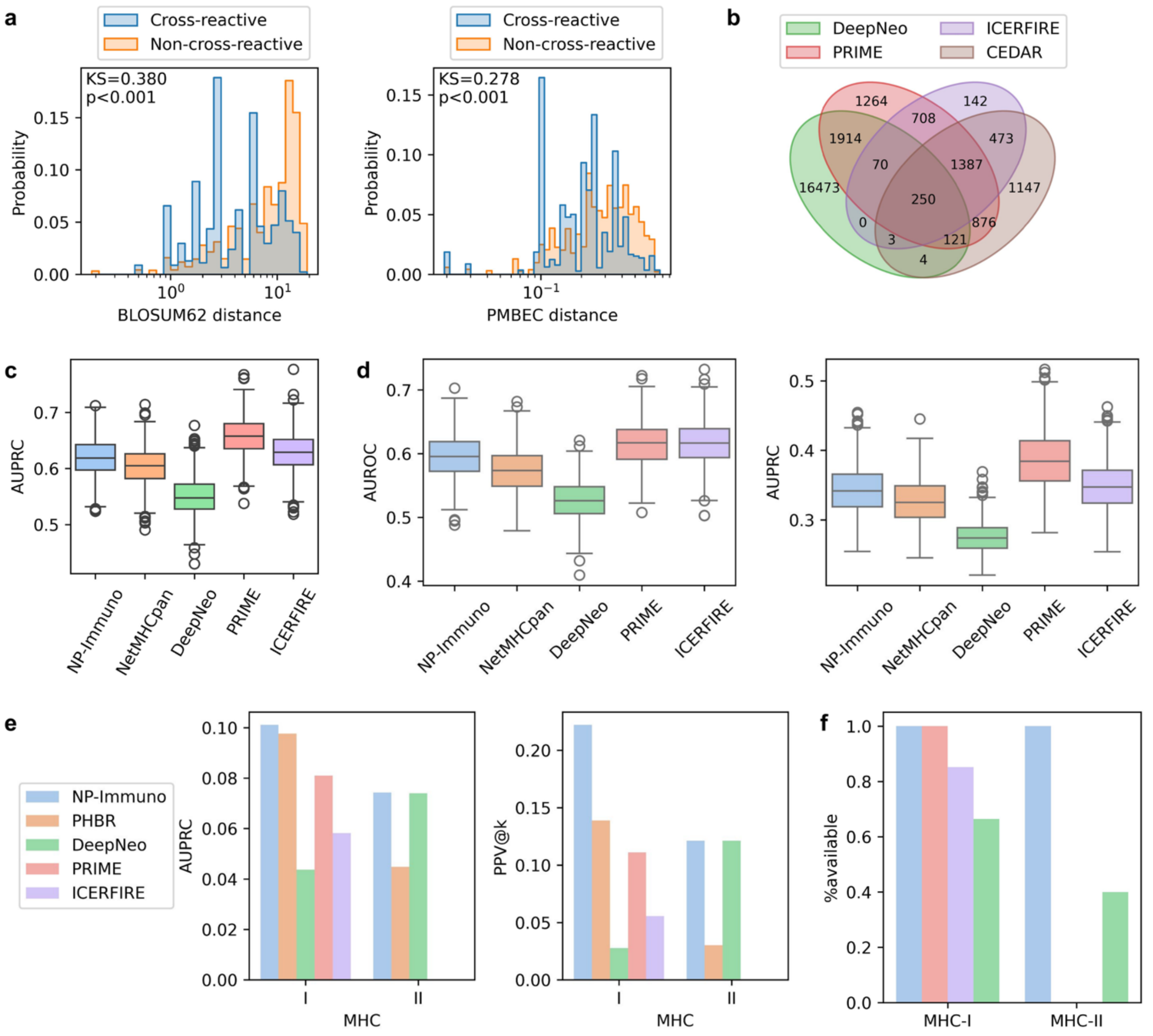


**Supplementary Fig. 2. Validation of NeoPrecis (NP)-Immuno.** **a** Distribution of BLOSUM62 and PMBEC distances in the cross-reactive peptide triplet validation set. The cross-reactive group (n = 3,535) consists of pairs of seed and cross-reactive peptides that mimic non-immunogenic substitutions, while the non-cross-reactive group (n = 3,535) consists of pairs of seed and non-cross-reactive peptides that mimic immunogenic substitutions. Statistical comparisons were performed using a one-sided Kolmogorov-Smirnov test (BLOSUM62: KS statistic = 0.380, $P = 6.04 \times 10^{-228}$; PMBEC: KS statistic = 0.278, $P = 2.99 \times 10^{-121}$). **b** Venn diagram showing the overlap between the CEDAR dataset, PRIME training set, ICEFIRE training set, and DeepNeo training set for MHC-I. **c** Area under the precision-recall curve (AUPRC) for each predictor on MHC-I samples (n = 438) from the internal testing set (CEDAR dataset). AUPRC distributions were calculated using bootstrapping (1,000 iterations). In the boxplots, the center line represents the median, boxes indicate the interquartile range (IQR; 25th–75th percentile), and whiskers extend to the most extreme data points within 1.5× the IQR. Outliers are depicted as individual circles. NP denotes NeoPrecis. **d** AUROC and AUPRC for each predictor on rebalanced MHC-I samples (n = 438; positive-to-negative ratio adjusted to 1:3 through bootstrapping). Performance distributions were calculated using bootstrapping (1,000 iterations). **e** AUPRC and positive predictive value (PPV) at top k (k is the number of true positives; 36 for MHC-I and 33 for MHC-II) comparisons of each predictor on MHC-I (n = 1,089) and MHC-II (n = 1,189) samples from the external testing set (NCI dataset). **f** Allele availability across predictors in the NCI cohort. The y-axis represents the proportion of alleles covered by each predictor.

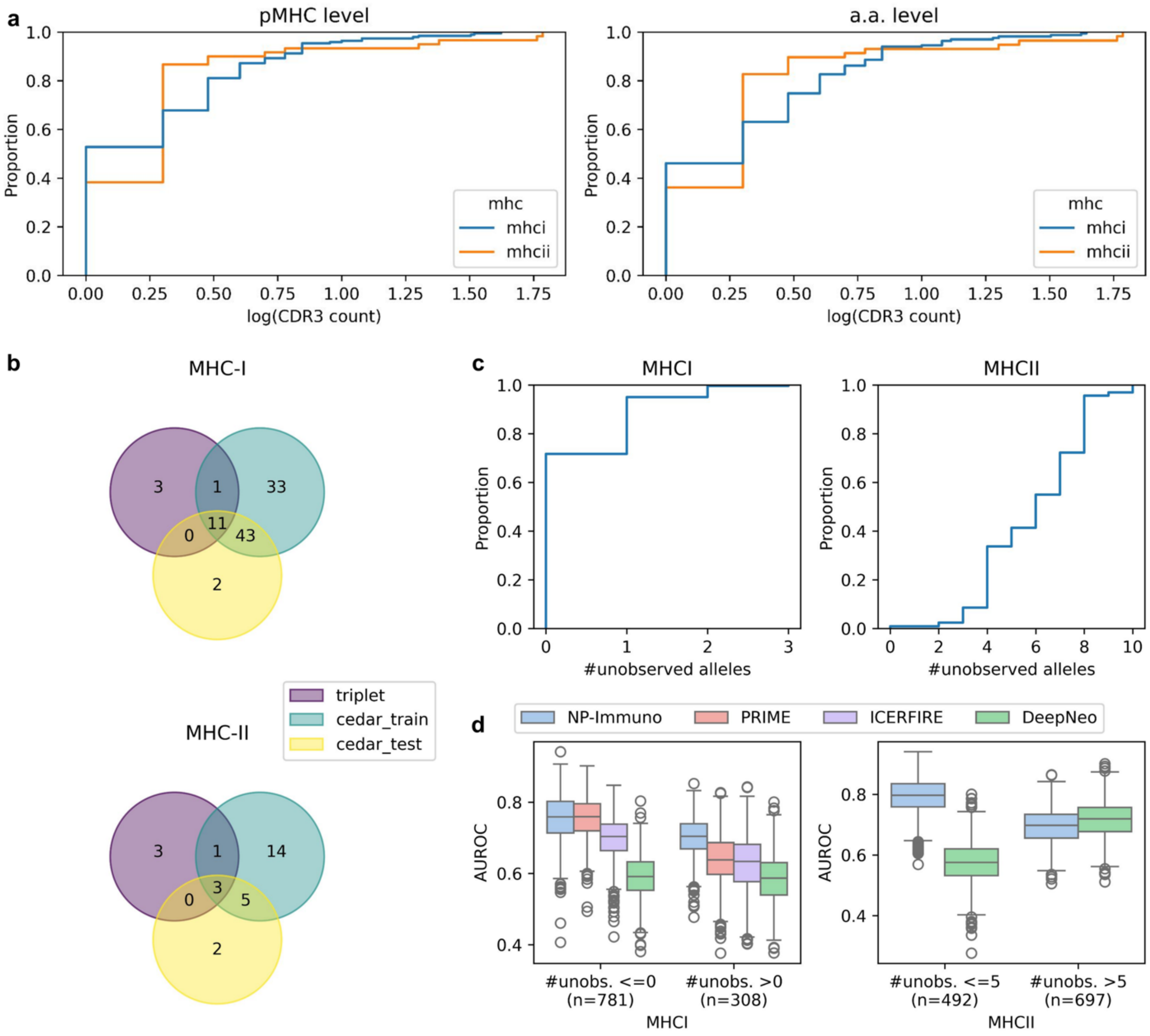


**Supplementary Fig. 3. Generalizability evaluation of NeoPrecis (NP)-Immuno.** **a** Cumulative distribution of the number of distinct CDR3 sequences supporting each cross-reactive peptide pair (left) and each amino acid substitution (right) in the triplet dataset. **b** Venn diagram showing allele overlap between the cross-reactive triplet dataset, CEDAR training set, and CEDAR testing set for MHC-I (top) and MHC-II (bottom). **c** Cumulative distribution of the number of unobserved alleles per mutation in the NCI dataset for MHC-I (left) and MHC-II (right). Unobserved alleles are those absent from the NeoPrecis-Immuno training data. **d** Performance (AUROC) of immunogenicity predictors stratified by unobserved allele groups for MHC-I (left) and MHC-II (right). Mutations were grouped by the number of unobserved alleles (0 vs. > 0 for MHC-I; ≤ 5 vs. > 5 for MHC-II). AUROC distributions were calculated using bootstrapping (1,000 iterations). In the boxplots, the center line represents the median, boxes indicate the interquartile range (IQR; 25th–75th percentile), and whiskers extend to the most extreme data points within 1.5× the IQR. Outliers are depicted as individual circles.

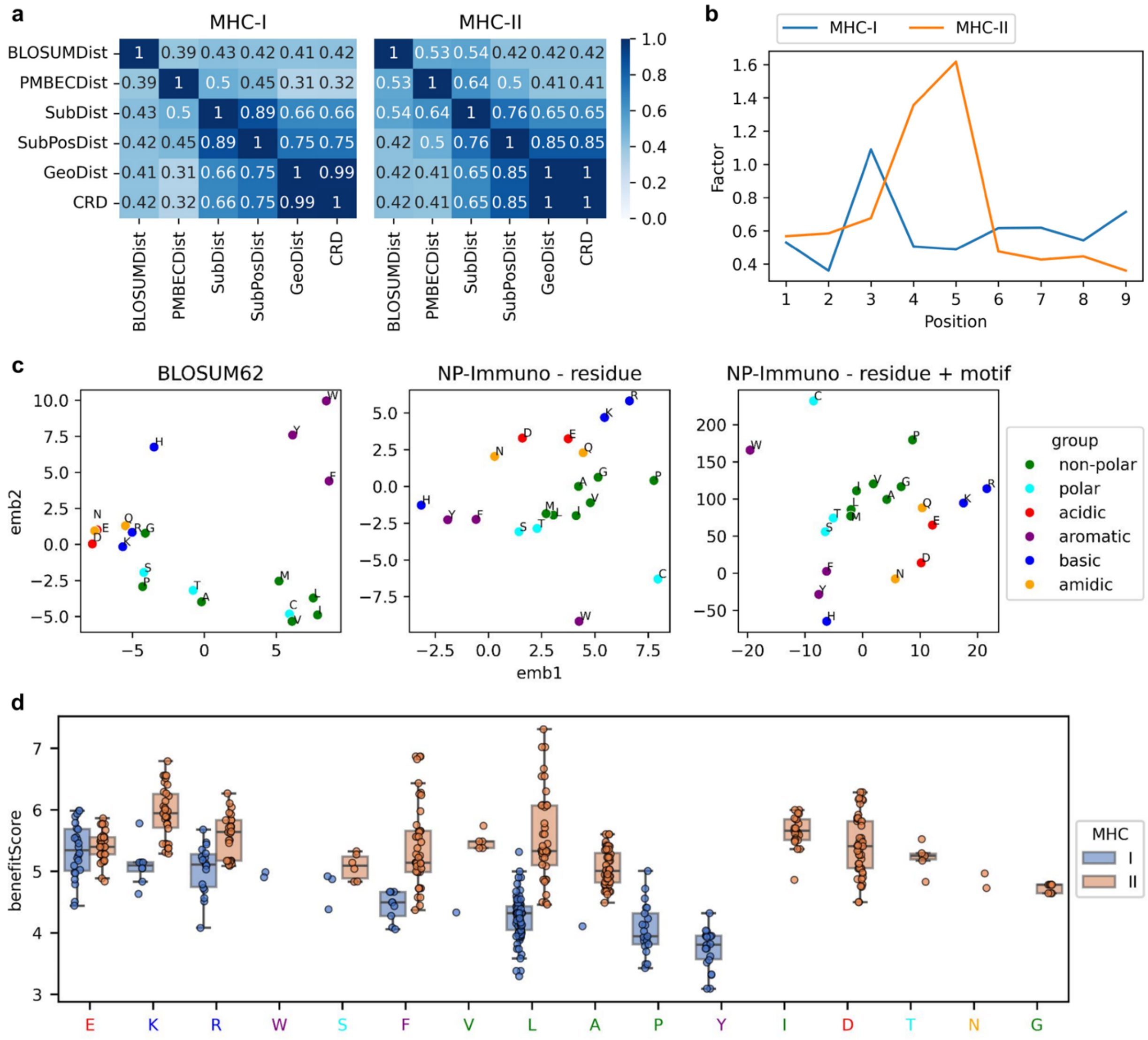

**Supplementary Fig. 4. Interpretation of NeoPrecis (NP)-Immuno. a** Correlation between components for MHC-I and MHC-II. **b** Position factors for MHC-I and MHC-II. **c** Residue embedding transformation from BLOSUM62, model embedding, to motif-enriched embedding, illustrated using the second position of allele B*40:01 as an example. **d** Distribution of allele benefit scores, with alleles grouped by the dominant amino acid in the anchor motif. In the boxplots (shown for groups with n ≥ 5), the center line represents the median, boxes indicate the interquartile range (IQR; 25th–75th percentile), and whiskers extend to the most extreme data points within 1.5× the IQR. Individual data points are overlaid for all groups.

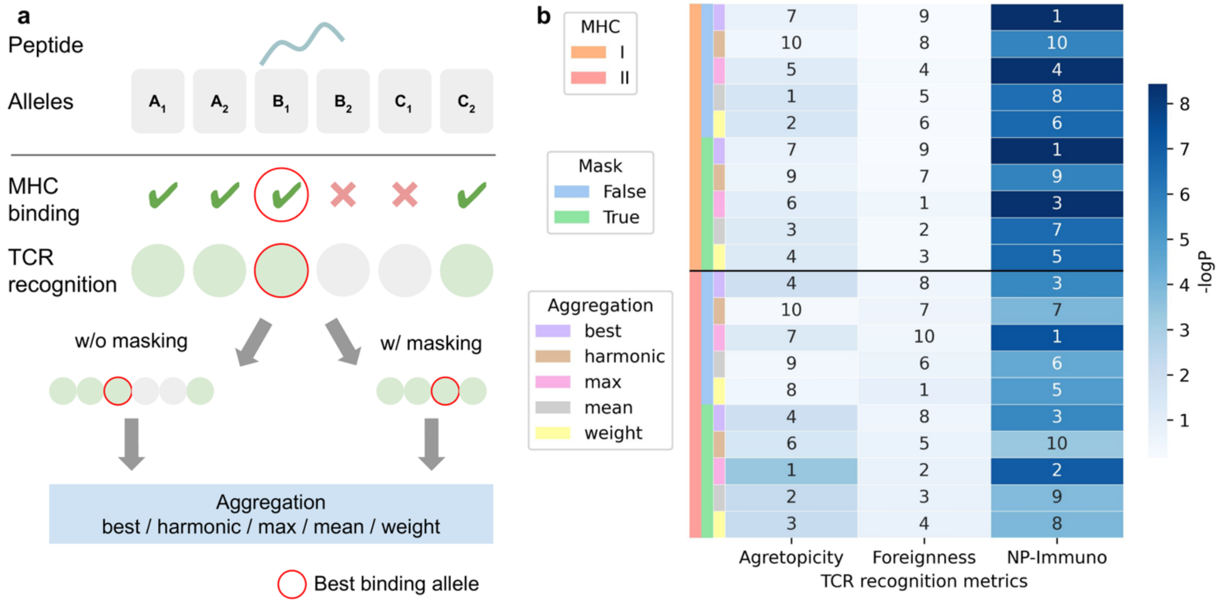


**Supplementary Fig. 5. Comparison of various aggregation approaches. a** The schematic illustrates the allele aggregation process. First, MHC-binding and TCR recognition metrics are calculated for each allele, and the best-binding allele is identified. In the masking approach, alleles without MHC binding are excluded, whereas in the non-masking approach, all alleles are retained. Various aggregation methods are then applied: "Best" represents the recognition score of the best-binding allele, "harmonic" represents the harmonic mean of recognition scores, "max" represents the highest value, "mean" represents the average, and "weight" represents the weighted average, with weights based on binding affinity. **b** The heatmap displays the negative log p-values from the association tests (one-sided Mann–Whitney U test) between each approach and T-cell activity across various TCR recognition metrics. Annotations indicate the rank within each MHC class and for each metric.

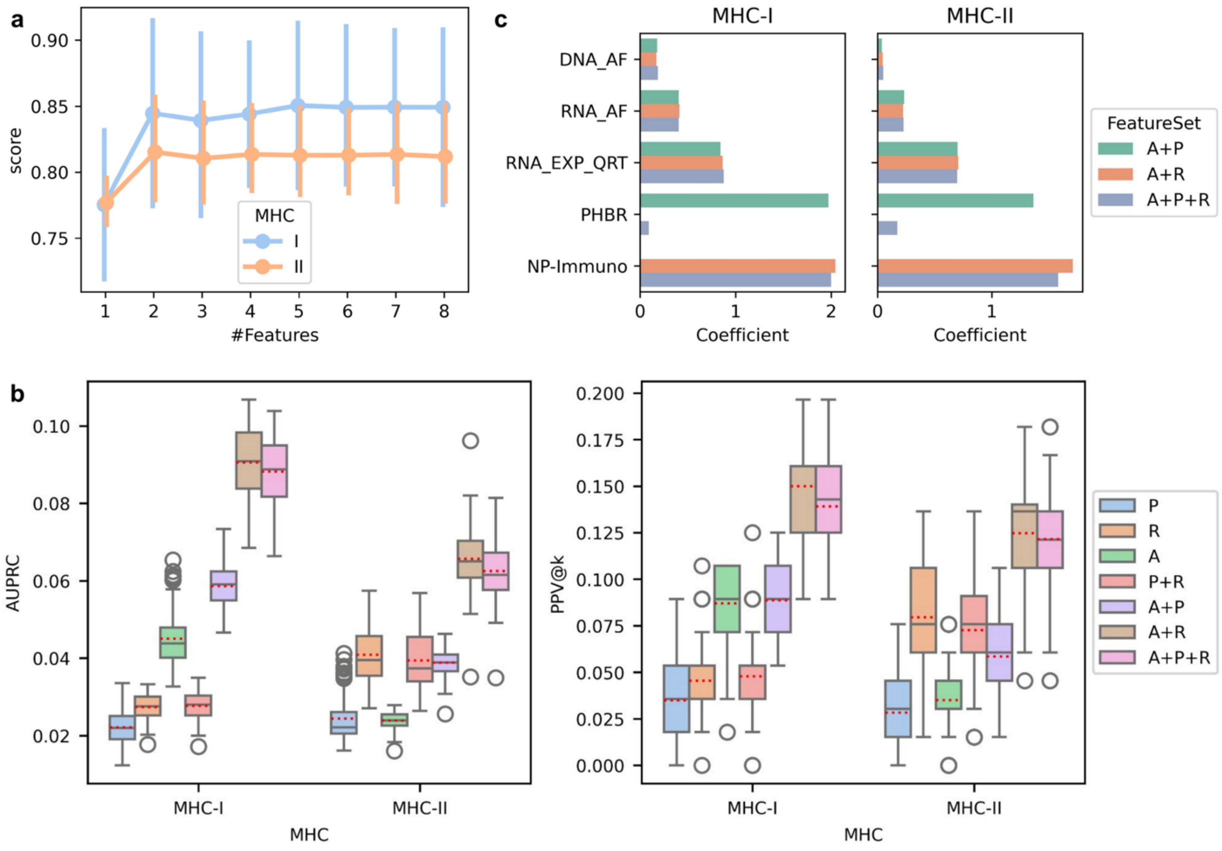


**Supplementary Fig. 6. Feature selection and performance evaluation of NeoPrecis-Integrated. a** Results of recursive feature elimination with cross-validation (RFECV) for selecting features for MHC-I and MHC-II. Points represent the mean AUROC of a 4-fold cross-validation (n = 4). Error bars indicate the 95% confidence interval. **b** AUPRC and PPV at top k (k = 56 for MHC-I; k = 66 for MHC-II) for 4-fold cross-validation repeated 100 times on the NCI cohort using different combinations of features. In the boxplots, the center line represents the median, boxes indicate the interquartile range (IQR; 25th–75th percentile), and whiskers extend to the most extreme data points within 1.5× the IQR. Outliers are depicted as individual circles. The mean is indicated by a red dashed line. **c** Feature importance in the integrated models using feature sets A+P, A+R, and A+P+R for MHC-I (left) and MHC-II (right). Features were standardized prior to modeling, and importance values are derived from logistic regression coefficients.

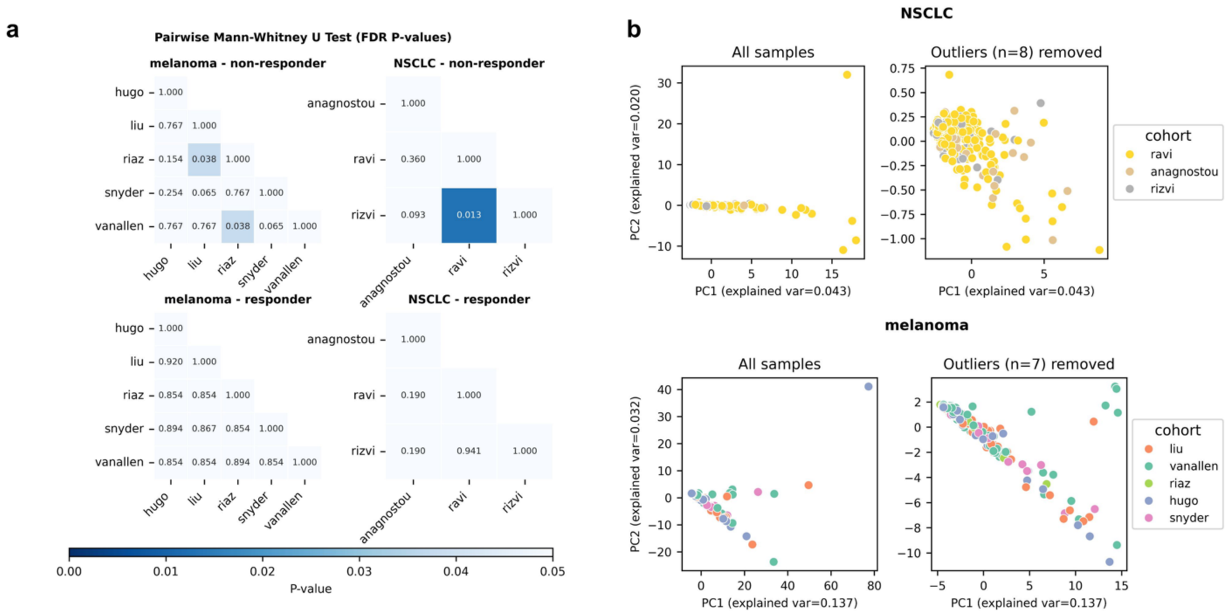


**Supplementary Fig. 7. Batch effect evaluation across ICI cohorts. a** Pairwise two-sided Mann-Whitney U test comparing TMB distributions across cohorts within the same cancer type and response category for melanoma (left) and NSCLC (right). Heatmaps show FDR-corrected p-values. **b** Distribution of top 2 principal components of binary mutation profiles for NSCLC (top) and melanoma (bottom). Each point represents a patient, colored by cohort.

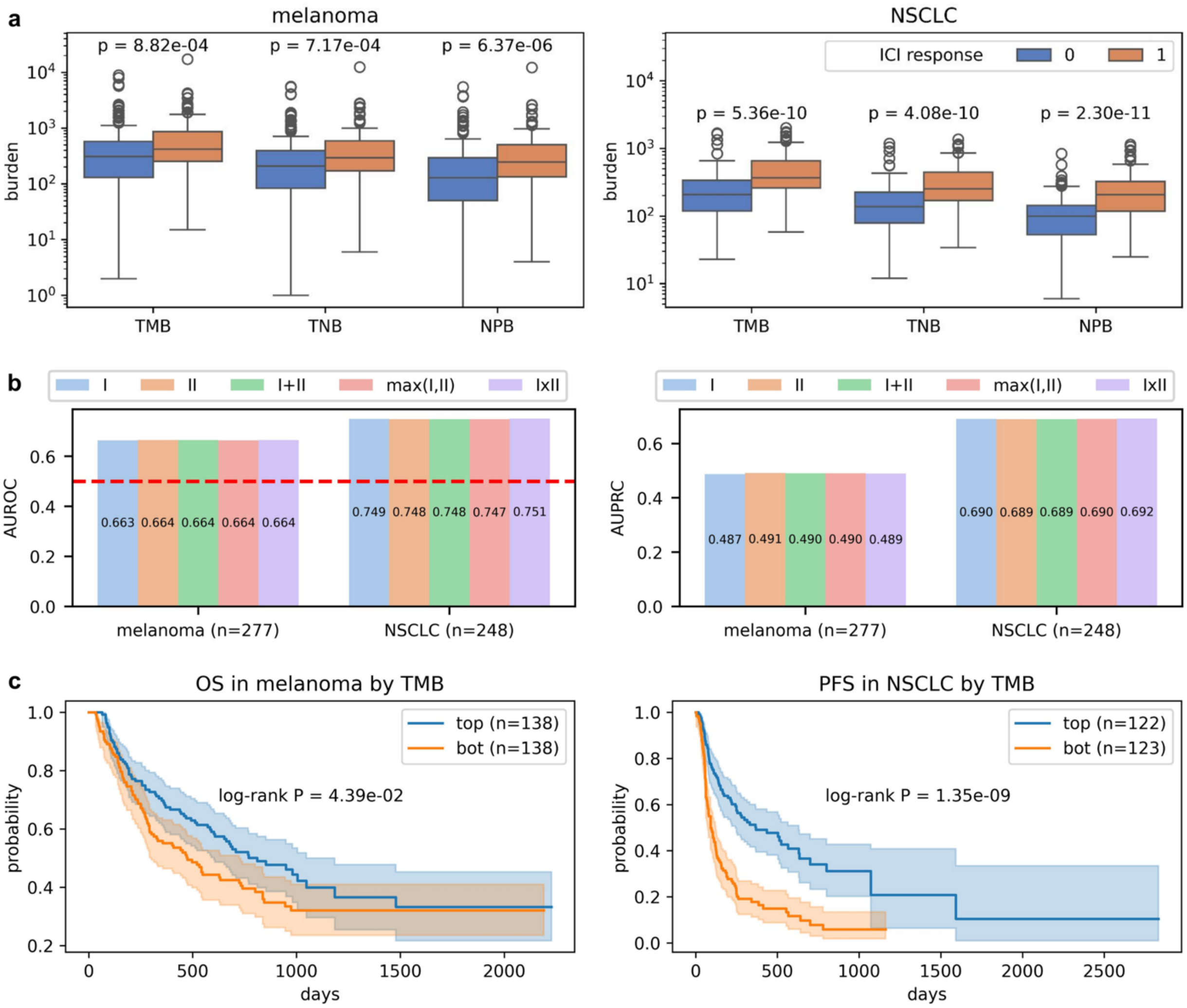


**Supplementary Fig. 8. Performance evaluation of NeoPrecis on ICI response prediction. a** Comparison of mutation burden distributions for melanoma and NSCLC. P-values were calculated using the two-sided Mann-Whitney U test. In the boxplots, the center line represents the median, boxes indicate the interquartile range (IQR; 25th–75th percentile), and whiskers extend to the most extreme data points within 1.5× the IQR. Outliers are depicted as individual circles. **b** Comparison of NeoPrecis-LandscapeSum scores using MHC-I-only, MHC-II-only, or MHC-dual (combined MHC-I and MHC-II). For MHC-dual, three aggregation methods were compared: multiplication (I×II), summation (I+II), and maximum (max(I,II)). **c** Kaplan-Meier survival curves stratified by TMB, with log-rank P-values shown for melanoma and NSCLC. Shaded areas indicate the 95% confidence interval.

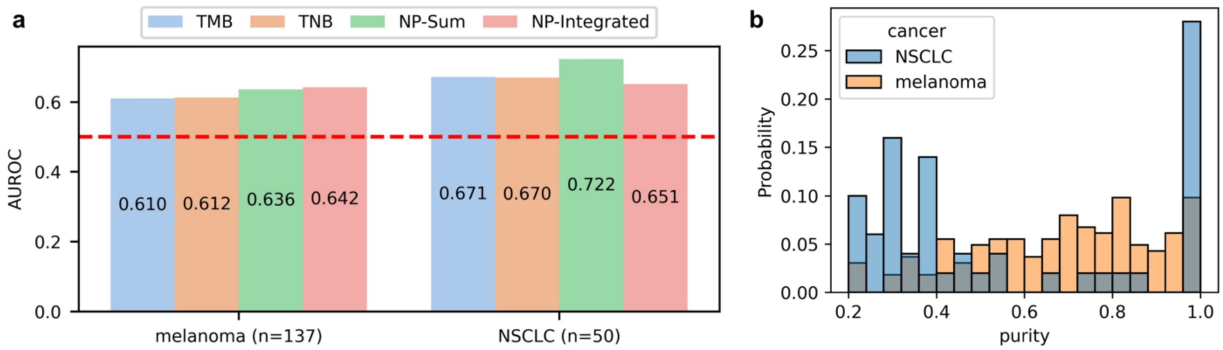


**Supplementary Fig. 9. Performance evaluation of multi-dimensional integration on RNA-available samples. a** AUROC comparison of TMB, TNB, NeoPrecis-LandscapeSum (NP-Sum), and NeoPrecis-Integrated (NP-Integrated) on RNA-available samples (n = 187; 137 melanoma and 50 NSCLC). **b** Purity distribution of RNA-available samples in melanoma and NSCLC.

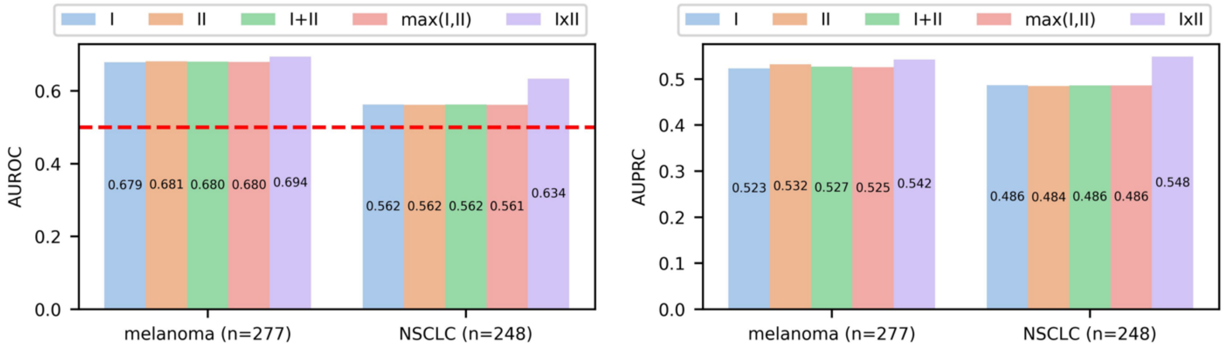


**Supplementary Fig. 10. Performance of subclone-level combination of MHC-I and MHC-II immunogenicity.** Comparison of NeoPrecis-LandscapeClone scores using MHC-I-only, MHC-II-only, or MHC-dual (combined MHC-I and MHC-II). For MHC-dual, three aggregation methods were compared: multiplication (I×II), summation (I+II), and maximum (max(I,II)).

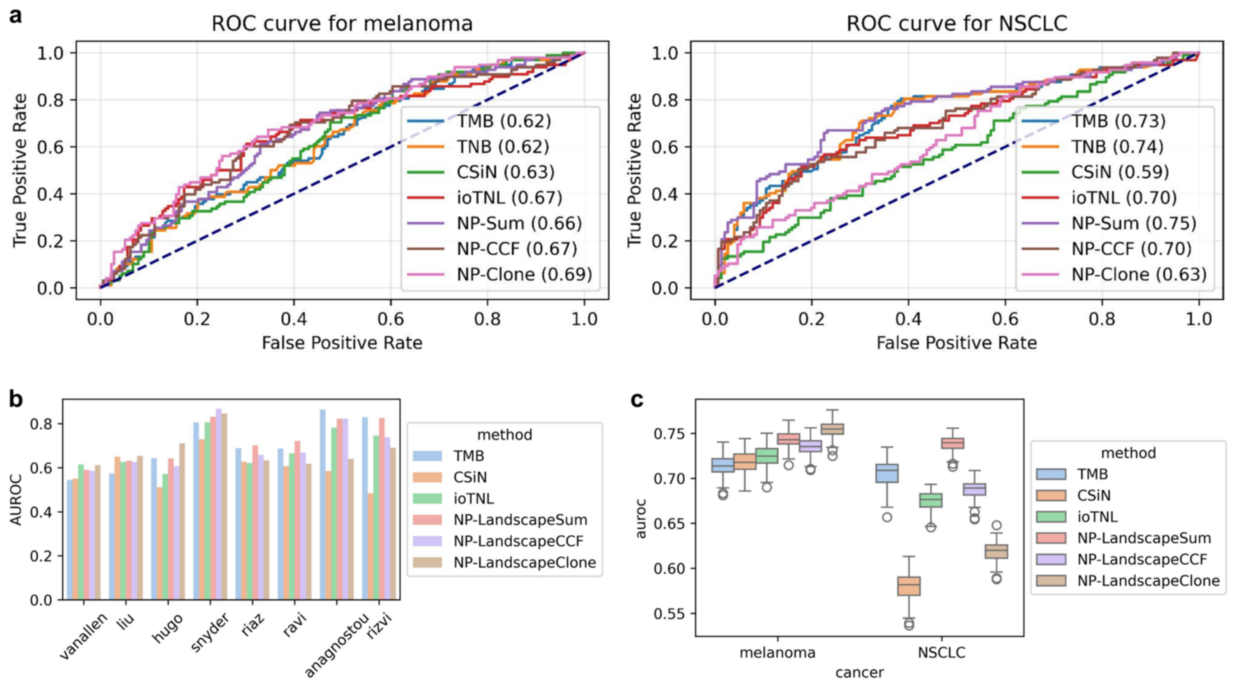


**Supplementary Fig. 11. Performance of tumor-centric immunogenicity scores in melanoma and NSCLC. a** AUROC comparison of tumor-centric immunogenicity metrics, including TMB, TNB, CSiN, ioTNL, NeoPrecis-LandscapeSum (NP-Sum), NeoPrecis-LandscapeCCF (NP-CCF), and NeoPrecis-LandscapeClone (NP-Clone), for response prediction in melanoma and NSCLC. **b** Cohort-specific AUROC performance comparing TMB, CSiN, ioTNL, NP-LandscapeSum, NP-LandscapeCCF and NP-LandscapeClone within individual cohorts. **c** AUROC performance using 5-fold cross-validation repeated 100 times with cohort incorporated as a covariate for melanoma (left) and NSCLC (right). In the boxplots, the center line represents the median, boxes indicate the interquartile range (IQR; 25th–75th percentile), and whiskers extend to the most extreme data points within 1.5× the IQR. Outliers are depicted as individual circles.

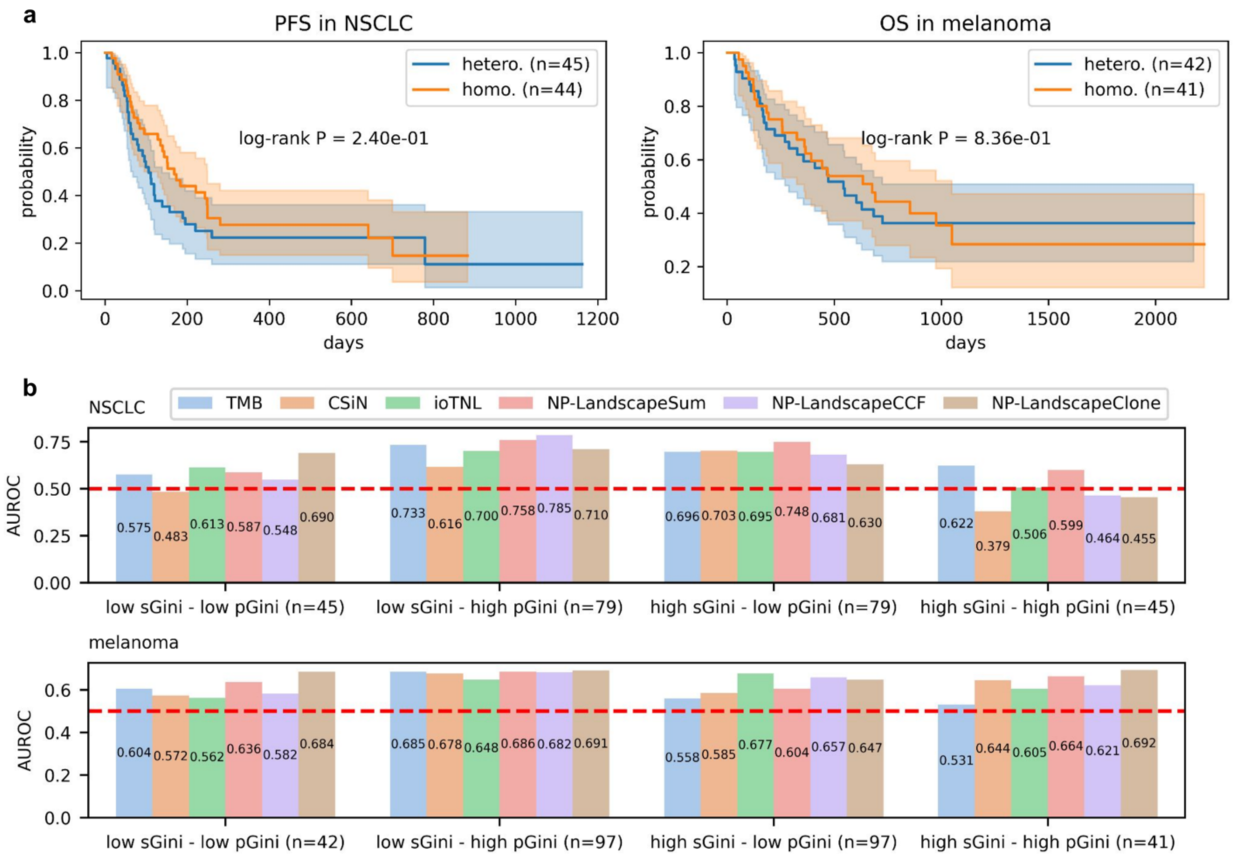


**Supplementary Fig. 12. Clonality analysis in melanoma and NSCLC. a** Survival curves comparing homogeneous (homo.; high sGini–high pGini) and heterogeneous (hetero.; low sGini–low pGini) tumor groups in melanoma (OS) and NSCLC (PFS). Shaded areas indicate the 95% confidence interval. **b** AUROC comparison of TMB, CSiN, ioTNL, NP-LandscapeSum, NP-LandscapeCCF, NP-LandscapeClone across four patient subgroups in melanoma and NSCLC. NP denotes NeoPrecis.

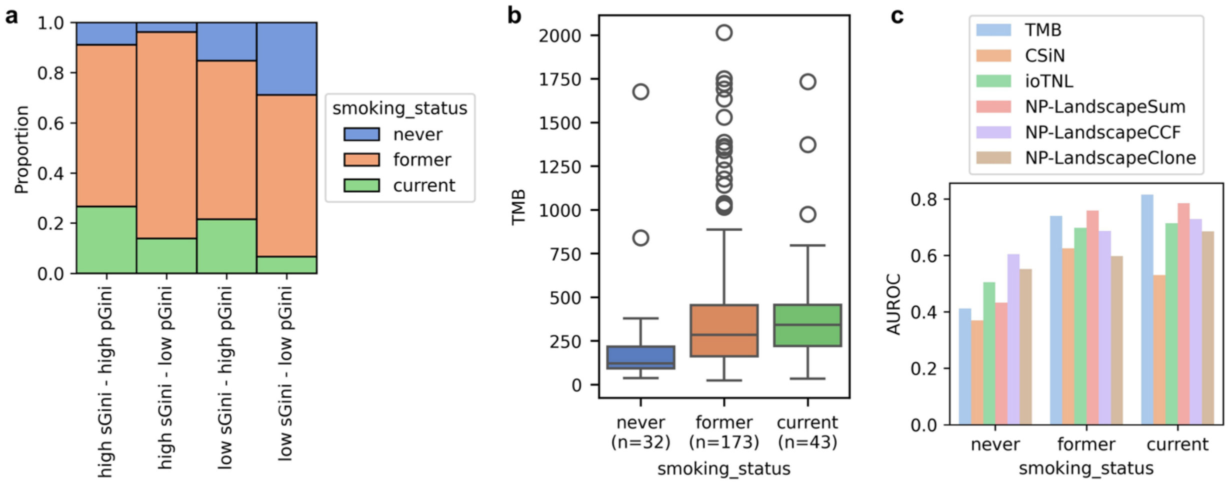


**Supplementary Fig. 13. Smoking status in NSCLC. a** Distribution of smoking status across four heterogeneity-defined subgroups. **b** TMB distribution by smoking status. In the boxplots, the center line represents the median, boxes indicate the interquartile range (IQR; 25th–75th percentile), and whiskers extend to the most extreme data points within 1.5× the IQR. Outliers are depicted as individual circles. **c** AUROC comparison of TMB, CSiN, ioTNL, NP-LandscapeSum, NP-LandscapeCCF, and NP-LandscapeClone across different smoking statuses. NP denotes NeoPrecis.

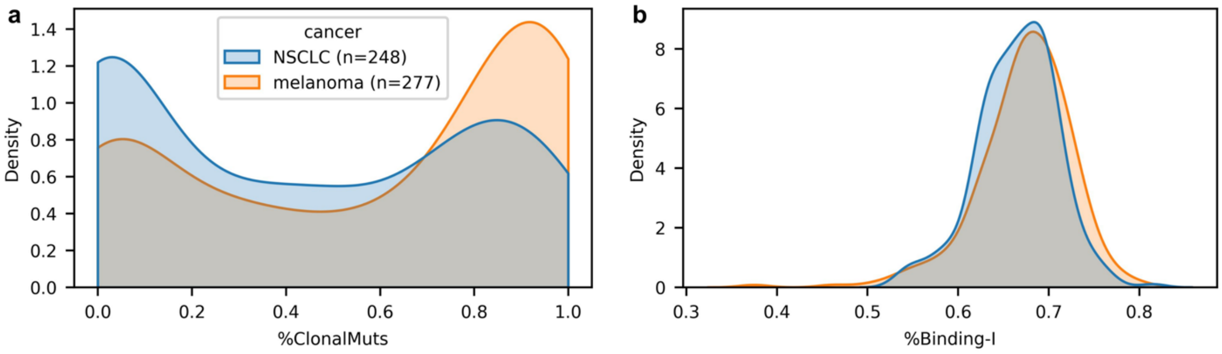


**Supplementary Fig. 14. Immunoediting analysis across cancer types. a** Density plot estimated using KDE showing the distribution of %ClonalMuts, the ratio of clonal mutations (CCF ≥ 0.85) to total mutations, across melanoma and NSCLC. Values are clipped between 0 and 1. **b** Density plot estimated using KDE showing the distribution of %Binding-I, the ratio of MHC-I binding mutations (PHBR ≤ 2) to total mutations, across melanoma and NSCLC. Values are clipped between 0 and 1.

## Supplementary Tables

| | Prior embedding | Residue embedding | Position factors | Motif enrichment | Sigmoid scaling |
|---|---|---|---|---|---|
| BLOSUMDist | v | | | | |
| SubDist | | v | | | |
| SubPosDist | | v | v | | |
| GeoDist | | v | v | v | |
| CRD | | v | v | v | v |

**Supplementary Table 1. Components of the NeoPrecis immunogenicity model.** To evaluate the contribution of individual components to NeoPrecis-Immuno, we assessed model performance by systematically including or excluding key features: residue embedding, position factors, motif enrichment, and sigmoid scaling. BLOSUM62 embedding served as a prior embedding for NeoPrecis. The rows of the table represent the different model configurations evaluated, while the columns indicate the presence or absence of each key component in a given model.

| | Cancer | #Samples | #RNA | #Response |
|---|---|---|---|---|
| Hugo et al. | melanoma | 38 | 27 | 21 |
| Van Allen *et al.* | melanoma | 110 | 40 | 17 |
| Riaz et al. | melanoma | 109 | 84 | 24 |
| Snyder *et al.* | melanoma | 64 | 0 | 36 |
| Liu et al. | melanoma | 122 | 122 | 50 |
| Rizvi *et al.* | NSCLC | 34 | 0 | 12 |
| Anagnostou *et al.* | NSCLC | 36 | 0 | 17 |
| Ravi et al. | NSCLC | 182 | 51 | 68 |

**Supplementary Table 2. Meta data of the ICI cohorts.** Each row in this table represents a cancer cohort from a specific study, annotated with the cancer type (Cancer), the total number of patients (#Samples), the number of patients with RNA-seq data available (#RNA), and the number of patients who responded to immune checkpoint inhibitors (#Response).
