# Supplementary material for: NeoPrecis: enhancing immunotherapy response prediction through integration of qualified immunogenicity and clonality-aware neoantigen landscapes
Source: Nat Commun. 2026 Jan 23;17:1966. doi: 10.1038/s41467-026-68651-6 (PMC12932759; doi:10.1038/s41467-026-68651-6)
Supplement: Supplementary file 2 — Description of Additional Supplementary Files [file 41467_2026_68651_MOESM2_ESM.pdf]

Description of Additional Supplementary Files

Supplementary Data 1. Dataset of cross-reactive peptide triplets.

Each row in this dataset represents a cross-reactive peptide triplet, comprising a seed peptide, a cross-reactive peptide, and a non-cross-reactive peptide. The seed and cross-reactive peptides are single Hamming distance variants that bind to the same MHC molecule and TCR CDR3 region, as identified from the TCR binding data obtained from IEDB and VDJdb. Non-cross-reactive peptides, each one Hamming distance from the seed but not present in the TCR binding data, were randomly selected. To ensure MHC binding, these non-cross-reactive peptides were subjected to binding prediction. For each cross-reactive pair, five non-cross-reactive peptides with the same mutated position and five with different mutated positions were sampled. In total, we produced 11,530 MHC-I and 2,610 MHC-II triplets.

Supplementary Data 2. CEDAR immunogenicity dataset.

Each row in this dataset represents a cancer-related mutant peptide with an immunogenicity label verified by T-cell assays, derived from the CEDAR dataset. For both wild-type and mutated peptides, MHC binding predictions were calculated. Additionally, immunogenicity predictions from PRIME, DeepNeo, ICERFIRE, and NeoPrecis-Immuno were included, along with an annotation indicating their presence in each predictor's training set.

Supplementary Data 3. NCI gastrointestinal cancer cohort dataset.

This dataset, derived from Parkhurst et al., includes 7,384 mutations (6,952 missense mutations) across 75 patients. Abundance annotations (DNA allelic fraction (DNA_AF), RNA allelic fraction (RNA_AF), and RNA expression quartile level (RNA_EXP_QRT)) and immunogenicity labels (CD4 and CD8 T-cell activations) were obtained from the original study. For each mutation, we calculated MHC presentation metrics (robustness and PHBR) and T-cell recognition metrics (agretopicity ratio, foreignness, and NeoPrecis-Immuno). Additionally, benchmark scores from PRIME, DeepNeo, and ICERFIRE were incorporated.

Supplementary Data 4. Allele benefit scores.

The benefit score, derived from the scaling factors of NeoPrecis-Immuno for each allele, is a mutation-independent metric representing the potential for enhanced tumor immunity associated with that allele.

Supplementary Data 5. Immune checkpoint inhibitor cohort dataset.

Each row in this dataset represents a patient, with metadata including age, sex, ICI response, and survival data. Mutation burden, CSiN, ioTNL, NeoPrecis prediction, and clonality analysis results are also annotated for each patient.
