# Supplementary material for: NeoPrecis: enhancing immunotherapy response prediction through integration of qualified immunogenicity and clonality-aware neoantigen landscapes
Source: Nat Commun. 2026 Jan 23;17:1966. doi: 10.1038/s41467-026-68651-6 (PMC12932759; doi:10.1038/s41467-026-68651-6)
Supplement: Supplementary file 8 — Reporting Summary [file 41467_2026_68651_MOESM8_ESM.pdf]

nature portfolio

Corresponding author(s): Hannah Carter

Last updated by author(s): Dec 17, 2025

Reporting Summary

Nature Portfolio wishes to improve the reproducibility of the work that we publish. This form provides structure for consistency and transparency in reporting. For further information on Nature Portfolio policies, see our Editorial Policies and the Editorial Policy Checklist.

Statistics

For all statistical analyses, confirm that the following items are present in the figure legend, table legend, main text, or Methods section.

n/a	Confirmed	
☐	☒	The exact sample size (*n*) for each experimental group/condition, given as a discrete number and unit of measurement
☐	☒	A statement on whether measurements were taken from distinct samples or whether the same sample was measured repeatedly
☐	☒	The statistical test(s) used AND whether they are one- or two-sided<br>*Only common tests should be described solely by name; describe more complex techniques in the Methods section.*
☐	☒	A description of all covariates tested
☐	☒	A description of any assumptions or corrections, such as tests of normality and adjustment for multiple comparisons
☐	☒	A full description of the statistical parameters including central tendency (e.g. means) or other basic estimates (e.g. regression coefficient) AND variation (e.g. standard deviation) or associated estimates of uncertainty (e.g. confidence intervals)
☐	☒	For null hypothesis testing, the test statistic (e.g. *F*, *t*, *r*) with confidence intervals, effect sizes, degrees of freedom and *P* value noted<br>*Give P values as exact values whenever suitable.*
☒	☐	For Bayesian analysis, information on the choice of priors and Markov chain Monte Carlo settings
☐	☒	For hierarchical and complex designs, identification of the appropriate level for tests and full reporting of outcomes
☐	☒	Estimates of effect sizes (e.g. Cohen's *d*, Pearson's *r*), indicating how they were calculated

Our web collection on statistics for biologists contains articles on many of the points above.

Software and code

Policy information about availability of computer code

Data collection	TCR-peptide binding data were collected from IEDB and VDJdb database. Genomic data were collected from Sequence Read Archive (SRA) using SRA-toolkit (v3.0.7).
Data analysis	Variant calling was performed using Nextflow-Sarek (v3.4.2), employing Mutect2 for somatic variant calling, VEP for mutation annotation, and ASCAT for copy number analysis, tumor purity, and ploidy estimation. RNA sequencing data were aligned to hg38 using STAR (v2.7.3a), and transcript quantification was conducted using RSEM (v1.3.1). HLA typing was performed with HLA-HD (v1.7.0). Clonality analysis was conducted using PyClone (v0.13.1). MHC-binding prediction was performed with NetMHCpan-4.1 and NetMHCIIpan-4.3.

For manuscripts utilizing custom algorithms or software that are central to the research but not yet described in published literature, software must be made available to editors and reviewers. We strongly encourage code deposition in a community repository (e.g. GitHub). See the Nature Portfolio guidelines for submitting code & software for further information.

Data

Policy information about availability of data

All manuscripts must include a data availability statement. This statement should provide the following information, where applicable:

- Accession codes, unique identifiers, or web links for publicly available datasets
- A description of any restrictions on data availability
- For clinical datasets or third party data, please ensure that the statement adheres to our policy

The processed data generated in this study are provided in the Supplementary Data files, including cross-reactive peptide triplets, CEDAR immunogenicity data, processed NCI gastrointestinal cancer cohort data, and the ICI cohort metadata with analysis results. Publicly available datasets analyzed in this study were obtained from the Immune Epitope Database (IEDB, https://www.iedb.org), VDJdb (https://vdjdb.cdr3.net), and the Cancer Epitope Database and Analysis Resource (CEDAR, https://cedar.iedb.org). The mutation-centric immunogenicity data (NCI dataset) were obtained from the supplementary materials of Parkhurst et al. (https://doi.org/10.1158/2159-8290.CD-18-1494). Clinical and genomic data from the reanalyzed ICI cohorts were obtained with the following accession numbers: Hugo et al. (SRP090294 [https://trace.ncbi.nlm.nih.gov/Traces/study/?acc=SRP090294], SRP067938 [https://trace.ncbi.nlm.nih.gov/Traces/study/?acc=SRP067938]), Van Allen et al. (SRP011540 [https://trace.ncbi.nlm.nih.gov/Traces/study/?acc=SRP011540]), Snyder et al. (SRP072934 [https://trace.ncbi.nlm.nih.gov/Traces/study/?acc=SRP072934]), Riaz et al. (SRP094781 [https://trace.ncbi.nlm.nih.gov/Traces/study/?acc=SRP094781]), Liu et al. (SRP011540 [https://trace.ncbi.nlm.nih.gov/Traces/study/?acc=SRP011540]), Ravi et al. (SRP413932 [https://trace.ncbi.nlm.nih.gov/Traces/study/?acc=SRP413932]), Anagnostou et al. (SRP238904 [https://trace.ncbi.nlm.nih.gov/Traces/study/?acc=SRP238904]), Rizvi et al. (SRP064805 [https://trace.ncbi.nlm.nih.gov/Traces/study/?acc=SRP064805]). Codes for conducting immunogenicity prediction and neoantigen landscape evaluation are deposited at both GitHub (https://github.com/cartercompbio/NeoPrecis) and Zenodo (https://doi.org/10.5281/zenodo.17959604).

Research involving human participants, their data, or biological material

Policy information about studies with human participants or human data. See also policy information about sex, gender (identity/presentation), and sexual orientation and race, ethnicity and racism.

Reporting on sex and gender	Sex was included as a covariate in the analysis of Cox proportional hazards model.
Reporting on race, ethnicity, or other socially relevant groupings	Race, ethnicity, or other socially relevant groupings are not considered in this study.
Population characteristics	Information is available in the supplementary data 5.
Recruitment	Immunotherapy cohorts were collected from Hugo et al. (SRP090294, SRP067938), Van Allen et al. (SRP011540), Snyder et al. (SRP072934), Riaz et al. (SRP094781), Liu et al. (SRP011540), Ravi et al. (SRP413932), Anagnostou et al. (SRP238904), Rizvi et al. (SRP064805). This research focused on two cancer types, melanoma and non-small cell lung cancer. Data were selected based on the availability of sequencing data and clinical outcomes.
Ethics oversight	Not applicable. This study is a reanalysis of existing deidentified data.

Note that full information on the approval of the study protocol must also be provided in the manuscript.

Field-specific reporting

Please select the one below that is the best fit for your research. If you are not sure, read the appropriate sections before making your selection.

☒ Life sciences ☐ Behavioural & social sciences ☐ Ecological, evolutionary & environmental sciences

For a reference copy of the document with all sections, see nature.com/documents/nr-reporting-summary-flat.pdf

Life sciences study design

All studies must disclose on these points even when the disclosure is negative.

Sample size	Five melanoma and three non-small cell lung cancer (NSCLC) cohorts were collected in this study. In total 695 patients, including 443 melanoma and 252 NSCLC), with whole-exome sequencing data were collected from dbGaP.
Data exclusions	Patients with pre-treatment biopsies and no prior ICI treatment were included. One sample that failed clonal analysis and nine samples lacking RECIST labels were excluded, resulting in 525 patients (277 melanoma and 248 NSCLC).
Replication	All the analyses are replicable with the provided code.
Randomization	Not applicable. All samples were used in evaluating the model performance.
Blinding	Not applicable. This is a retrospective analysis and all samples collected were used.

Reporting for specific materials, systems and methods

We require information from authors about some types of materials, experimental systems and methods used in many studies. Here, indicate whether each material, system or method listed is relevant to your study. If you are not sure if a list item applies to your research, read the appropriate section before selecting a response.

Materials & experimental systems

n/a	Involved in the study	
☒	☐	Antibodies
☒	☐	Eukaryotic cell lines
☒	☐	Palaeontology and archaeology
☒	☐	Animals and other organisms
☐	☒	Clinical data
☒	☐	Dual use research of concern
☒	☐	Plants

Methods

n/a	Involved in the study	
☒	☐	ChIP-seq
☒	☐	Flow cytometry
☒	☐	MRI-based neuroimaging

Clinical data

Policy information about clinical studies

All manuscripts should comply with the ICMJE guidelines for publication of clinical research and a completed CONSORT checklist must be included with all submissions.

Clinical trial registration	No clinical trial was conducted in this study.
Study protocol	*Note where the full trial protocol can be accessed OR if not available, explain why.*
Data collection	*Describe the settings and locales of data collection, noting the time periods of recruitment and data collection.*
Outcomes	*Describe how you pre-defined primary and secondary outcome measures and how you assessed these measures.*

Plants

Seed stocks	*Report on the source of all seed stocks or other plant material used. If applicable, state the seed stock centre and catalogue number. If plant specimens were collected from the field, describe the collection location, date and sampling procedures.*
Novel plant genotypes	*Describe the methods by which all novel plant genotypes were produced. This includes those generated by transgenic approaches, gene editing, chemical/radiation-based mutagenesis and hybridization. For transgenic lines, describe the transformation method, the number of independent lines analyzed and the generation upon which experiments were performed. For gene-edited lines, describe the editor used, the endogenous sequence targeted for editing, the targeting guide RNA sequence (if applicable) and how the editor was applied.*
Authentication	*Describe any authentication procedures for each seed stock used or novel genotype generated. Describe any experiments used to assess the effect of a mutation and, where applicable, how potential secondary effects (e.g. second site T-DNA insertions, mosiacism, off-target gene editing) were examined.*
